# Diaqua­(2,2′-bipyridine-6,6′-dicarboxyl­ato)nickel(II)

**DOI:** 10.1107/S1600536811016400

**Published:** 2011-05-07

**Authors:** Shui Hu, ShiPeng Wen, Huai-Ming Hu, Li Liu

**Affiliations:** a Key Laboratory of Carbon Fiber and Functional Polymer, Ministry of Education, Beijing University of Chemical Technology, Beijing 100029, People’s Republic of China; b College of Chemistry and Materials Science, Northwest University, Xi’an 710069, People’s Republic of China

## Abstract

In the title compound, [Ni(C_12_H_6_N_2_O_4_)(H_2_O)_2_], the Ni^II^ atom (site symmetry 2) displays a distorted *cis*-NiN_2_O_4_ octa­hedral coordination geometry with two N atoms and two O atoms of the tetra­dentate 2,2′-bipyridine-6,6′-dicarboxyl­ate ligand in the equatorial plane and two water mol­ecules in axial positions. The complete dianionic ligand is generated by crystallographic twofold symmetry. In the crystal, a two-dimensional supra­molecular structure parallel to (001) is formed through O—H⋯O hydrogen-bond inter­actions between the coordinated water mol­ecules and the O atoms of nearby carboxyl­ate groups.

## Related literature

For transition metal complexes with the title ligand, see: Knight *et al.* (2006 ▶); Duan *et al.* (2009 ▶); Wang *et al.* (2009 ▶). For lanthanide metal complexes with the title ligand, see: Bunzli *et al.* (2000 ▶); Wang *et al.* (2010 ▶).
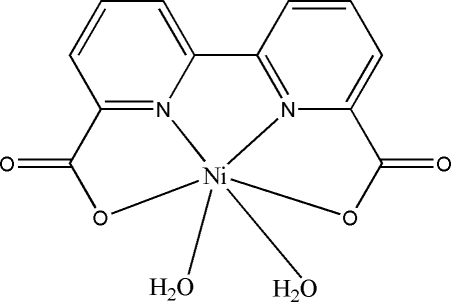


## Experimental

### 

#### Crystal data

[Ni(C_12_H_6_N_2_O_4_)(H_2_O)_2_]
*M* *_r_* = 336.93
Orthorhombic, 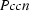

*a* = 7.1056 (9) Å
*b* = 11.3608 (15) Å
*c* = 15.3334 (19) Å
*V* = 1237.8 (3) Å^3^
*Z* = 4
Mo *K*α radiation
μ = 1.60 mm^−1^
*T* = 296 K
0.24 × 0.16 × 0.10 mm

#### Data collection

Bruker APEXII CCD diffractometer
Absorption correction: multi-scan (*SADABS*; Bruker, 2005 ▶) *T*_min_ = 0.766, *T*_max_ = 0.857
6269 measured reflections
1274 independent reflections
1098 reflections with *I* > 2σ(*I*)
*R*_int_ = 0.036

#### Refinement

*R*[*F* ^2^ > 2σ(*F* ^2^)] = 0.032
*wR*(*F* ^2^) = 0.082
*S* = 1.06
1274 reflections
102 parameters
2 restraints
H atoms treated by a mixture of independent and constrained refinement
Δρ_max_ = 0.48 e Å^−3^
Δρ_min_ = −0.23 e Å^−3^

### 

Data collection: *APEX2* (Bruker, 2005 ▶); cell refinement: *SAINT* (Bruker, 2005 ▶); data reduction: *SAINT*; program(s) used to solve structure: *SHELXS97* (Sheldrick, 2008 ▶); program(s) used to refine structure: *SHELXL97* (Sheldrick, 2008 ▶); molecular graphics: *SHELXTL* (Sheldrick, 2008 ▶); software used to prepare material for publication: *SHELXTL*.

## Supplementary Material

Crystal structure: contains datablocks I, global. DOI: 10.1107/S1600536811016400/hb5864sup1.cif

Structure factors: contains datablocks I. DOI: 10.1107/S1600536811016400/hb5864Isup2.hkl

Supplementary material file. DOI: 10.1107/S1600536811016400/hb5864Isup3.cdx

Additional supplementary materials:  crystallographic information; 3D view; checkCIF report

## Figures and Tables

**Table 1:** Selected bond lengths (Å)

Ni1—N1	1.9975 (19)
Ni1—O3	2.0553 (18)
Ni1—O1	2.1335 (16)

**Table 2:** Hydrogen-bond geometry (Å, °)

*D*—H⋯*A*	*D*—H	H⋯*A*	*D*⋯*A*	*D*—H⋯*A*
O3—H3*A*⋯O2^i^	0.81 (2)	1.90 (2)	2.708 (2)	176 (3)
O3—H3*B*⋯O2^ii^	0.83 (2)	1.95 (2)	2.772 (3)	172 (3)

Symmetry codes: (i) 
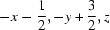
; (ii) 
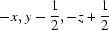
.

**Fig. 1.**
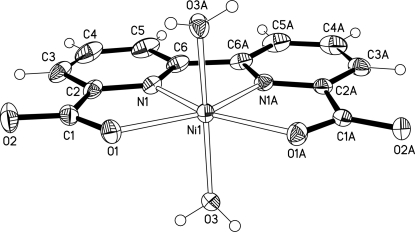
The molecular structure of (I), showing 30% probability displacement ellipsoids. Atoms labelled with the suffix A are at the symmetry position (-x + 1/2, -y + 3/2, z).

**Fig. 2.**
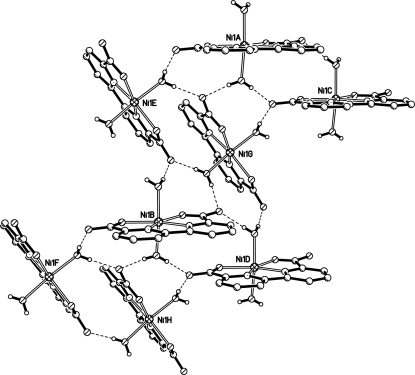
View of a two-dimensional supramolecular structure constructed through hydrogen bonding interactions in (I). Hydrogen atoms of carbon atoms have been omitted for clarity.

## supplementary crystallographic information

### Comment

Pyridyl carboxylic acid is an important class of organic ligands and has been widely used in coordination chemistry. 2,2'-Bipyridine-6,6'-dicarboxylate ligand is coordinated woth transition metal (Duan *et al.*, 2009; Knight *et al.*, 2006 and Wang *et al.*, 2009) and lanthanide metal ions (Bunzli *et al.*, 2000 and Wang *et al.*, 2010). Herein, we report crystal structure of a new nickel complex with 2,2'-bipyridine-6,6'-dicarboxylate ligand.

The atom-numbering scheme of (I) is shown in Fig. 1. The Ni^II^ atom displays a distorted octahedral coordination geometry with two N atoms and two O atoms of 2,2'-bipyridine-6,6'-dicarboxylate in equatorial plane and two water molecules in apical positions. A two-dimensional supramolecular structure is formed through hydrogen interactions between the oxygen atoms of coordination water molecules and the oxygen atoms of carboxylate groups [O3—H3A···O2^i^, 2.708 (3) Å, 176 (3) °, symmetric code i: (-*x* - 1/2, -*y* + 3/2, *z*); O3—H3B···O2^ii^, 2.772 (3) Å, 172 (3) °, symmetric code ii: (-*x*, *y* - 1/2, -*z* + 1/2)].

### Experimental

The title compound was prepared by the reaction of Ni(NO_3_)_2_ with 2,2'-bipyridine-6,6'-dicarboxylic acid (H_2_bpdc) in a water solution. Ni(NO_3_)_2_.6H_2_O (0.2 mmol) and H_2_bpdc (0.2 mmol) were dissolved in 25 ml deionized water and adjusted the pH to 7 with 0.05 mol *L*^-1^ NaOH aqueous solution. After one week, green blocks were obtained. Elmental analysis for C_12_H_10_N_2_NiO_6_ calculated: C 42.78, H 2.99, N 8.32%; found: C 42.57, H 2.89, N 8.46%.

### Refinement

The water H atoms were located in a difference Fourier map and refined with restrained O—H bond lengths [0.85 (2) Å] and fixed isotropic displancement parameters (*U*_iso_(H) = 1.2 *U*_eq_(O)). The carbon H atoms were placed at calculated positions (C—H = 0.93–0.96 Å) and refined as riding model with *U*_iso_(H) = 1.2 *U*_eq_(carrier).

### Crystal data

**Table:**

[Ni(C_12_H_6_N_2_O_4_)(H_2_O)_2_]	*F*(000) = 688
*M**_r_* = 336.93	*D*_x_ = 1.808 Mg m^−^^3^
Orthorhombic, *P**c**c**n*	Mo *K*α radiation, λ = 0.71073 Å
Hall symbol: -P 2ab 2ac	Cell parameters from 1650 reflections
*a* = 7.1056 (9) Å	θ = 3.2–25.2°
*b* = 11.3608 (15) Å	µ = 1.60 mm^−^^1^
*c* = 15.3334 (19) Å	*T* = 296 K
*V* = 1237.8 (3) Å^3^	Block, green
*Z* = 4	0.24 × 0.16 × 0.10 mm

### Data collection

**Table:**

Bruker APEXII CCD diffractometer	1274 independent reflections
Radiation source: fine-focus sealed tube	1098 reflections with *I* > 2σ(*I*)
graphite	*R*_int_ = 0.036
φ and ω scans	θ_max_ = 26.4°, θ_min_ = 2.7°
Absorption correction: multi-scan (*SADABS*; Bruker, 2005)	*h* = −8→8
*T*_min_ = 0.766, *T*_max_ = 0.857	*k* = −13→14
6269 measured reflections	*l* = −10→19

### Refinement

**Table:**

Refinement on *F*^2^	Primary atom site location: structure-invariant direct methods
Least-squares matrix: full	Secondary atom site location: difference Fourier map
*R*[*F*^2^ > 2σ(*F*^2^)] = 0.032	Hydrogen site location: inferred from neighbouring sites
*wR*(*F*^2^) = 0.082	H atoms treated by a mixture of independent and constrained refinement
*S* = 1.06	*w* = 1/[σ^2^(*F*_o_^2^) + (0.0405*P*)^2^ + 0.5888*P*] where *P* = (*F*_o_^2^ + 2*F*_c_^2^)/3
1274 reflections	(Δ/σ)_max_ < 0.001
102 parameters	Δρ_max_ = 0.48 e Å^−^^3^
2 restraints	Δρ_min_ = −0.23 e Å^−^^3^

### Special details

**Table:**

Geometry. All e.s.d.'s (except the e.s.d. in the dihedral angle between two l.s. planes) are estimated using the full covariance matrix. The cell e.s.d.'s are taken into account individually in the estimation of e.s.d.'s in distances, angles and torsion angles; correlations between e.s.d.'s in cell parameters are only used when they are defined by crystal symmetry. An approximate (isotropic) treatment of cell e.s.d.'s is used for estimating e.s.d.'s involving l.s. planes.
Refinement. Refinement of *F*^2^ against ALL reflections. The weighted *R*-factor *wR* and goodness of fit *S* are based on *F*^2^, conventional *R*-factors *R* are based on *F*, with *F* set to zero for negative *F*^2^. The threshold expression of *F*^2^ > σ(*F*^2^) is used only for calculating *R*-factors(gt) *etc*. and is not relevant to the choice of reflections for refinement. *R*-factors based on *F*^2^ are statistically about twice as large as those based on *F*, and *R*- factors based on ALL data will be even larger.

### Fractional atomic coordinates and isotropic or equivalent isotropic displacement parameters (Å^2^)

**Table:**

	*x*	*y*	*z*	*U*_iso_*/*U*_eq_	
Ni1	0.2500	0.7500	0.16327 (3)	0.02540 (16)	
N1	0.0931 (3)	0.80137 (16)	0.06211 (12)	0.0285 (4)	
O1	0.0167 (2)	0.83346 (15)	0.22531 (11)	0.0360 (4)	
O2	−0.2446 (2)	0.93408 (18)	0.19595 (16)	0.0517 (6)	
C1	−0.1035 (3)	0.8756 (2)	0.17424 (18)	0.0348 (6)	
C2	−0.0683 (4)	0.8569 (2)	0.07707 (17)	0.0332 (6)	
C3	−0.1808 (4)	0.8936 (2)	0.0083 (2)	0.0464 (7)	
H3	−0.2934	0.9332	0.0182	0.056*	
C4	−0.1204 (5)	0.8695 (3)	−0.0750 (2)	0.0563 (9)	
H4	−0.1951	0.8915	−0.1221	0.068*	
C5	0.0486 (5)	0.8132 (2)	−0.09017 (18)	0.0506 (8)	
H5	0.0894	0.7982	−0.1467	0.061*	
C6	0.1564 (4)	0.7796 (2)	−0.01856 (16)	0.0348 (6)	
O3	0.1216 (2)	0.58888 (16)	0.17647 (13)	0.0380 (5)	
H3A	0.009 (3)	0.584 (3)	0.1803 (18)	0.046*	
H3B	0.169 (4)	0.545 (2)	0.2135 (15)	0.046*	

### Atomic displacement parameters (Å^2^)

**Table:**

	*U*^11^	*U*^22^	*U*^33^	*U*^12^	*U*^13^	*U*^23^
Ni1	0.0254 (3)	0.0293 (3)	0.0215 (2)	0.00250 (16)	0.000	0.000
N1	0.0341 (11)	0.0256 (10)	0.0260 (11)	−0.0004 (9)	−0.0053 (9)	−0.0005 (8)
O1	0.0336 (9)	0.0402 (10)	0.0343 (10)	0.0040 (8)	0.0046 (8)	−0.0040 (8)
O2	0.0263 (10)	0.0466 (12)	0.0821 (15)	0.0055 (8)	−0.0004 (9)	−0.0232 (11)
C1	0.0248 (12)	0.0269 (12)	0.0526 (17)	−0.0047 (10)	0.0005 (12)	−0.0078 (11)
C2	0.0316 (13)	0.0217 (12)	0.0464 (16)	−0.0021 (10)	−0.0109 (11)	−0.0021 (10)
C3	0.0437 (15)	0.0291 (14)	0.067 (2)	−0.0016 (11)	−0.0276 (15)	0.0050 (13)
C4	0.073 (2)	0.0378 (16)	0.059 (2)	−0.0073 (15)	−0.0407 (18)	0.0134 (14)
C5	0.085 (2)	0.0380 (16)	0.0289 (15)	−0.0094 (16)	−0.0177 (15)	0.0061 (11)
C6	0.0530 (17)	0.0261 (12)	0.0252 (12)	−0.0045 (11)	−0.0073 (12)	0.0018 (9)
O3	0.0259 (9)	0.0364 (10)	0.0519 (12)	0.0001 (8)	0.0044 (9)	0.0117 (8)

### Geometric parameters (Å, °)

**Table:**

Ni1—N1^i^	1.9975 (19)	C2—C3	1.387 (4)
Ni1—N1	1.9975 (19)	C3—C4	1.375 (5)
Ni1—O3^i^	2.0553 (18)	C3—H3	0.9300
Ni1—O3	2.0553 (18)	C4—C5	1.381 (5)
Ni1—O1^i^	2.1335 (16)	C4—H4	0.9300
Ni1—O1	2.1335 (16)	C5—C6	1.392 (4)
N1—C2	1.329 (3)	C5—H5	0.9300
N1—C6	1.339 (3)	C6—C6^i^	1.491 (5)
O1—C1	1.254 (3)	O3—H3A	0.806 (18)
O2—C1	1.247 (3)	O3—H3B	0.827 (17)
C1—C2	1.526 (4)		
			
N1^i^—Ni1—N1	78.11 (11)	O2—C1—C2	117.8 (2)
N1^i^—Ni1—O3^i^	95.10 (8)	O1—C1—C2	116.4 (2)
N1—Ni1—O3^i^	93.67 (8)	N1—C2—C3	120.6 (3)
N1^i^—Ni1—O3	93.67 (8)	N1—C2—C1	112.1 (2)
N1—Ni1—O3	95.10 (8)	C3—C2—C1	127.3 (3)
O3^i^—Ni1—O3	168.70 (11)	C4—C3—C2	117.8 (3)
N1^i^—Ni1—O1^i^	77.45 (7)	C4—C3—H3	121.1
N1—Ni1—O1^i^	155.48 (8)	C2—C3—H3	121.1
O3^i^—Ni1—O1^i^	90.40 (7)	C3—C4—C5	121.4 (3)
O3—Ni1—O1^i^	84.56 (7)	C3—C4—H4	119.3
N1^i^—Ni1—O1	155.48 (8)	C5—C4—H4	119.3
N1—Ni1—O1	77.45 (7)	C4—C5—C6	118.2 (3)
O3^i^—Ni1—O1	84.56 (7)	C4—C5—H5	120.9
O3—Ni1—O1	90.40 (7)	C6—C5—H5	120.9
O1^i^—Ni1—O1	127.04 (9)	N1—C6—C5	119.5 (3)
C2—N1—C6	122.5 (2)	N1—C6—C6^i^	112.53 (14)
C2—N1—Ni1	119.11 (17)	C5—C6—C6^i^	127.93 (19)
C6—N1—Ni1	118.41 (17)	Ni1—O3—H3A	121 (2)
C1—O1—Ni1	114.88 (15)	Ni1—O3—H3B	115 (2)
O2—C1—O1	125.7 (3)	H3A—O3—H3B	108 (3)
			
N1^i^—Ni1—N1—C2	−178.6 (2)	Ni1—N1—C2—C3	−179.86 (17)
O3^i^—Ni1—N1—C2	−84.19 (18)	C6—N1—C2—C1	−177.4 (2)
O3—Ni1—N1—C2	88.68 (18)	Ni1—N1—C2—C1	1.8 (3)
O1^i^—Ni1—N1—C2	176.75 (16)	O2—C1—C2—N1	175.2 (2)
O1—Ni1—N1—C2	−0.59 (17)	O1—C1—C2—N1	−2.6 (3)
N1^i^—Ni1—N1—C6	0.53 (13)	O2—C1—C2—C3	−3.1 (4)
O3^i^—Ni1—N1—C6	94.98 (18)	O1—C1—C2—C3	179.2 (2)
O3—Ni1—N1—C6	−92.15 (18)	N1—C2—C3—C4	0.6 (4)
O1^i^—Ni1—N1—C6	−4.1 (3)	C1—C2—C3—C4	178.7 (2)
O1—Ni1—N1—C6	178.57 (19)	C2—C3—C4—C5	−1.5 (4)
N1^i^—Ni1—O1—C1	3.7 (3)	C3—C4—C5—C6	1.0 (4)
N1—Ni1—O1—C1	−0.96 (16)	C2—N1—C6—C5	−1.6 (4)
O3^i^—Ni1—O1—C1	94.04 (17)	Ni1—N1—C6—C5	179.24 (19)
O3—Ni1—O1—C1	−96.09 (17)	C2—N1—C6—C6^i^	177.8 (2)
O1^i^—Ni1—O1—C1	−179.58 (17)	Ni1—N1—C6—C6^i^	−1.4 (3)
Ni1—O1—C1—O2	−175.4 (2)	C4—C5—C6—N1	0.6 (4)
Ni1—O1—C1—C2	2.1 (3)	C4—C5—C6—C6^i^	−178.7 (3)
C6—N1—C2—C3	1.0 (4)		

Symmetry codes: (i) −*x*+1/2, −*y*+3/2, *z*.

### Hydrogen-bond geometry (Å, °)

**Table:**

*D*—H···*A*	*D*—H	H···*A*	*D*···*A*	*D*—H···*A*
O3—H3A···O2^ii^	0.81 (2)	1.90 (2)	2.708 (2)	176 (3)
O3—H3B···O2^iii^	0.83 (2)	1.95 (2)	2.772 (3)	172 (3)

Symmetry codes: (ii) −*x*−1/2, −*y*+3/2, *z*; (iii) −*x*, *y*−1/2, −*z*+1/2.
